# Digital Luminescence Patterning via Inkjet Printing of a Photoacid Catalysed Organic-Inorganic Hybrid Formulation

**DOI:** 10.3390/polym11030430

**Published:** 2019-03-06

**Authors:** Jorge Alamán, María López-Valdeolivas, Raquel Alicante, Jose Ignacio Peña, Carlos Sánchez-Somolinos

**Affiliations:** 1 Departamento de Física de la Materia Condensada, Instituto de Ciencia de Materiales de Aragón (ICMA), CSIC-Universidad de Zaragoza, 50009 Zaragoza, Spain; jorge.alaman@bshg.com (J.A.); m_lopez@unizar.es (M.L.-V.); raquela@unizar.es (R.A.); 2 BSH Electrodomésticos España, S.A., Polígono Industrial de PLA-ZA, Ronda del Canal Imperial de Aragón, 18-20, 50197 Zaragoza, Spain; 3 Departamento de Ciencia y Tecnología de Materiales y Fluidos, Instituto de Ciencia de Materiales de Aragón (ICMA), CSIC-Universidad de Zaragoza, 50018 Zaragoza, Spain; jipena@unizar.es; 4 CIBER in Bioengineering, Biomaterials and Nanomedicine (CIBER-BBN), C Mariano Esquillor s.n., 50018 Zaragoza, Spain

**Keywords:** organic-inorganic hybrid materials, highly crosslinked polymers, photoacid generators, UV-photopolymerization, inkjet printing, luminescent materials

## Abstract

Accurate positioning of luminescent materials at the microscale is essential for the further development of diverse application fields including optoelectronics, energy, biotechnology and anti-counterfeiting. In this respect, inkjet printing has recently attracted great interest due to its ability to precisely deposit with high throughput and no contact, functional materials on different types of substrates. Here, we present a novel photoacid catalysed organic-inorganic hybrid luminescent ink. The formulation, containing monomers bearing epoxy and silane functionalities, a photoacid generator and a small percentage of Rhodamine-B, shows good jetting properties and adequate wetting of the deposited droplets on the receiving substrates. Ultraviolet exposure of the deposited material triggers the cationic ring-opening polymerization reaction of the epoxy groups. Concomitantly, if atmospheric water is available, hydrolysis and condensation takes place, overall leading to a luminescent crosslinked hybrid organic-inorganic polymeric material obtained through a simple one-step curing process, without post baking steps. Advantageously, protection of the ink from actinic light delays the hydrolysis and condensation conferring long-term stability to the ink. Digital patterning leads to patterned emissive surfaces and elements with good adhesion to different substrates, mechanical and optical properties for the fabrication of optical and photonic elements and devices.

## 1. Introduction

The development of luminescent systems is key for the further progress and expansion of a variety of application fields spanning from optoelectronics, solar energy harvesting and biotechnology to documents and goods anti-counterfeiting [1,2,3,4,5,6,7]. Light emissive centres typically used in these systems include organic fluorophores, organometallic luminescent molecules, rare earth ions or semiconductor quantum dots, many times incorporated in a solid matrix [8,9,10,11,12,13,14]. The selection of the type of luminescent entity and the host medium for each specific application responds to criteria of emission efficiency, emission wavelength and stability against temperature and illumination conditions, among other requirements. 

Inkjet printing has gained a lot of interest over the last years as a tool to create functional elements and devices [15,16,17,18,19,20] due to its ability to precisely deposit, with no contact, controlled-volume ink droplets on substrates of different nature, all this with high throughput despite being a serial process. In particular inkjet has been explored to generate luminescent patterns. Inks explored for this purpose need to incorporate luminescent entities, such as emissive chromophores or nanoparticles [21,22,23,24,25,26,27,28]. As inkjet is an additive printing method, all the deposited material, except for the solvents, is employed in the final device thus making an optimum use of these precious luminescent additives. Besides this, multiple materials can be sequentially added, enabling the integration of different functional elements and layers in the same device. This is of great potential for example in the formation of pixels in OLED displays, the preparation of sensor bioarrays or the printing of multifunctional security feature elements for documents and goods protection [29,30,31,32]. The development and optimization of inks require multiple variables to be considered [30,31]. As an example, appropriate ink viscosity and surface tension are critical to ensure jettability. Besides, adequate interaction between the ink and the target substrate is also needed to have well-defined prints. Many advanced applications require the precise microdeposition of the functional material onto a nonporous substrate [32,33,34,35,36]. Drying of the solvent usually leads to a concentration of the solid at the drop edge through the so called coffee ring effect, a phenomenon that can be, to a large extent, inhibited by a judicious selection of solvents [37,38]. Another approach to circumvent this problem is the use of phase change inks that quickly solidify after impact. Similarly, UV reactive inks, once deposited, can be fixed though polymerization when exposed to actinic light [36,37,39]. This last approach has been employed in the preparation of luminescent inks comprising acrylate or epoxy monomers together with emitting chromophores. After deposition, these are fixed through a light induced reaction leading to an organic polymeric material showing luminescent properties [21,24,40].

Besides these organic based matrix systems, hybrid materials encompass advantages from both, organic and inorganic materials [41]. Hybrid systems show good processability at low temperatures, by means of different patterning techniques. Additionally the final systems typically present good optical quality, tunability of their properties via the selection of proper monomers and their proportions, as well as excellent mechanical properties and adhesion to different types of substrates [42,43,44,45]. Additionally the inclusion of luminescent centres in hybrid hosts usually lead to more efficient emission as well as thermal and environmental stability of the light emitting entities [45]. Despite all these advantages, the inclusion of the luminescent functionality in hybrid formulations for inkjet has been scarcely studied and the preparation of luminescent curable hybrid systems that can be directly fixed after deposition remains nearly unexplored. As one of the few examples, the group of Valiyaveettil has recently demonstrated inkjet printing of thermally curable inks based on the polydimethylsiloxane elastomer kit Sylgard 184^®^ (Dow Corning Corporation, Midland, MI, USA) with luminescent silica nanoparticles dispersed on it [46]. In another example a sol-gel derived YAG:Er3+ precursor is used to generate, through inkjet printing, fine line patterns that, after firing of the samples, lead to miniaturized scintillators [47].

Recently we have demonstrated that photoacid catalysed formulations containing monomers with epoxy and silane functionalities are suitable systems for the preparation of inks with long-term stability leading, after printing and curing, to deposits with excellent optical and mechanical properties [48]. When compared to other sol-gel methods, this approach is a solvent-free process starting with the low molecular weight monomers that advantageously enable the preparation of formulations with appropriate viscosity for inkjet. The use of suitable photoinitiators in the formulation, allows to concomitantly cure the organic and inorganic network by irradiating with UV light [49,50,51]. The process is carried out at room temperature (RT) without the need of post baking steps. Additionally, in this approach, in comparison to conventional hybrid inks containing prehydrolysed–condensed reactive inorganic precursors [52,53], the hydrolysis and condensation beneficially takes place after the printing step, when the sample is irradiated with UV light. This favours the long-term stability of the ink properties (e.g., viscosity) needed to preserve its jettability and therefore its applicability in industrial applications. To further explore the potential of this polymerization reaction in the preparation of photocurable functional formulations for inkjet, we have incorporated the luminescent functionality in these reactive systems. Here we report a new light emitting, jettable, solvent-free ink, based on a photoacid catalysed organic-inorganic hybrid formulation, containing monomers with epoxy and silane functionalities and a luminescent dye. Rhodamine B, which has been successfully integrated in other hybrid matrices, has been chosen here as light emissive moiety [54,55,56,57,58,59,60]. After deposition, UV irradiation of the inks leads to the cationic ring-opening polymerization of epoxy functionalities and the hydrolysis and condensation of silanes. The resulting cured material presents excellent adhesion to glass and good mechanical properties without any further postprocessing step. Luminescent properties of the deposited materials after curing have been characterized too. To further leverage the potential of these photocurable systems, the use of this luminescent ink in the preparation of light emissive digital patterns on substrates of different nature has also been explored.

## 2. Materials and Methods 

### 2.1. Materials

The chemical structure of the molecules employed for the ink preparation is presented in Scheme S1 in the Supplementary Information. 3-Glycidoxypropyltrimethoxysilane (GPTMS), a hybrid organic-inorganic molecule provided with an epoxy and a trialkoxysilane, was acquired from Alfa Aesar (Haverhill, MA, USA). GPTMS is a low viscosity liquid at RT. The polymeric epoxy resin Epikote 157, that is a monomer having an average of eight aromatic benzene rings and eight epoxide groups, was purchased from Momentive (Waterford, NY, USA). Epikote 157 appears as flakes at RT. Dimethoxydiphenylsilane (dPDMS) is a liquid disilane monomer bearing two aromatic rings, from Aldrich. As a photoacid generator (PAG), triarylsulfonium hexafluorophosphate salts (50% in propylene carbonate), acquired from Aldrich (Madrid, Spain)., were used. This compound, when excited with UV actinic light, triggers the polymerization of the organic epoxide groups and, concurrently, act as a catalyst for the hydrolysis and condensation of the alkoxides. To regulate the surface tension of the ink, BYK-333 (BYK Chemie), a polyether-modified polydimethylsiloxane that also improves the surface wetting, was employed. To provide the ink with luminescent properties, 2-[6-(diethylamino)-3-(diethylimino)-3H-xanthen-9-yl] benzoic acid, usually known as Rhodamine B, was added to the formulation. Rhodamine B was purchased from Lambda Physic (Santa Clara, CA, USA). under the reference Lambdachrome LC6100. All the materials were used as received.

### 2.2. Experimental Procedures

#### 2.2.1. Ink Preparation

Photocurable luminescent formulations were prepared by directly mixing the above mentioned materials in the corresponding percentages. To facilitate homogenization, Epikote 157 was thoroughly grinded previously to its addition to the mixture. The mixtures were stirred at RT using a magnetic stirrer at 600 rpm until a homogeneous formulation was obtained.

#### 2.2.2. Substrates and Cleaning Procedure

Conventional microscope glass slides were employed as substrates. A pre-cleaning of these substrates was carried out using a solution of soapy water by gently hand rubbing the surface, using nitrile gloves. After thoroughly rinsing the substrates with water, they were introduced in an ultrasonic bath with soapy water for 10 min. After this, the substrates were refluxed with milli-Q water and ultrasonicated again in milli-Q water for 10 min. After cleaning with water, the substrates were refluxed with isopropyl alcohol (IPA) and a third ultrasonic bath was carried out, in this case with IPA for 10 min. Finally, the glass substrates were immediately dried with compressed air and stored until their use. Cyclic olefin polymer (COP) from Zeonor (188 μm thick polymer foil and microscope slides), uncoated poly(ethylene terephthalate) (PET) film as well as indium tin oxide (ITO) coated PET (220 μm thick polymer foil) from Aldrich were used as thermoplastic substrates for demonstrators. All these plastic substrates are provided with a protective foil that is removed just before printing.

#### 2.2.3. Substrate Treatments

UV Ozone treatment was performed on a UV-ozone reactor UVO 342 (Jelight company Inc., Irvine, CA, USA) to remove any remaining organic contamination of glass [61]. In some of the samples, a Pyrosil treatment (Pyrosil^®^, SURA, Jena, Germany), consisting in a combustion chemical vapor deposition (CCVD), was also carried out for the activation of the surfaces. An organosilicon precursor is injected into a gas flame that is put in short contact with the substrate, leading to a SiO_2_-like coating with thickness typically below 50–100 nm. As a result, important changes in wettability and adhesion are obtained [62]. 

#### 2.2.4. Inkjet Printing 

For the inkjet printing a custom-made inkjet printer system was used (In-2 Printing Solutions, Navarra, Spain) with Xaar-126/80 piezoelectric printheads (Xaar, Cambridge, UK). These printheads have 126 nozzles (50 μm diameter) arranged in a line with a pitch of 137 μm. The line of nozzles is perpendicular to the direction of the substrate motion, that moves under the fixed printhead. As a result, the vertical resolution (in the direction of the line of nozzles) is 185 dots per inch (dpi). The horizontal resolution (in the direction of the substrate movement) depends on different factors such as firing frequency and relative speed of the substrate with the printhead. The printhead is commanded by the Xaar XUSB drive electronics that is controlled with a PC and its corresponding software (from Xaar, Cambrdige, UK). This software enables a precise control of the parameters that command the printhead, detection of the samples and definition of the patterns to be printed (bitmap file). The movement of the substrate while printing takes usually place at constant speed of 20 mm/s by using an eTrack linear stage from Newmark systems Inc. (Mission Viejo, CA, USA) commanded by IMS-Terminal software (Marlborough, MA, USA). The printhead is mounted in a metallic block, provided with a heater and thermocouple connected to a temperature relay that regulates the temperature of the printhead at the desired set point. 

#### 2.2.5. UV-Curing Fixation 

An UV lamp Exfo OmniCure S2000 UV (Gentec, Nivelles, Belgium) has been used with an UV bandpass filter (wavelength range of 320–390 nm). To cure the films, a power of 10 mW/cm^2^ was applied during 5 min. Curing was carried out at RT (26 °C) either in an ambient atmosphere, with a relative humidity between 30% and 40% or under mild vacuum conditions. In this last case, the samples were cured inside a chamber provided with an optical access. A mild vacuum (100 mBar) can be attained inside the chamber by using a vacuum pump. Once the desired pressure level is achieved, UV exposure is immediately carried out to minimize evaporation of the deposited ink components. 

### 2.3. Characterization

#### 2.3.1. Ink Properties Characterization 

Viscosity of the ink was measured by using a Haake Rheostress 1 rotational Rheometer from Thermo Scientific, (Waltham, MA, USA). Surface tension was characterized using the pendant droplet method in an Attension Goniometer Theta Lite. The given surface tension values are the result of an average of three independent measurements. Density measurements were carried out using a 10 mL pycnometer. 

#### 2.3.2. Flying Droplet Characterization

Together with the printing system, a home-built dropwatcher was employed for the analysis of the characteristics of the ejected drops. This allows the characterization and optimization of the printing system configuration, especially parameters that fix the voltage sent to the printhead piezoelectric elements and so optimize the final drop characteristics. This visualization system consists on a CCD camera, a strobe led device and a pulse generator to synchronize the signal of the printhead, the strobe light and the camera acquisition events. 

#### 2.3.3. Deposited Ink and Film Characterization

Fourier Transform Infrared (FTIR) Spectroscopy was performed using a Perkin Elmer Spectrum 100 with ATR accessory. For FTIR spectra, thin films of the formulation were applied using a Meyer Rod bar nominally providing a wet film thickness of 24 μm. FTIR spectra were measured between 4000–450 cm^−1^. UV-Vis absorption measurements of the solutions and the films were carried out using a VARIAN Cary-500 spectrophotometer. Luminescent properties of the deposited films (emission and excitation spectra) were characterized using a Perkin Elmer LS50B spectrometer. Optical microscope images of the deposited drops were taken using an optical microscope OLYMPUS Eclipse i80. A Field-Emission Scanning electron microscope (FE-SEM) Merlin Carl Zeiss (Oberkochen, Germany) was used to study the morphology of the films. Hardness and elastic module of the films were measured by nanoindentation, using a Nanoindenter G200 from Agilent Technologies (Santa Clara, CA, USA) equipped with a Vickers indenter tip. Values were calculated on the basis of 4 indentations in 2 identical samples. Adhesion of the cured films to the glass substrate was characterized using a cross-cut and tape test according to the ASTM D3359 standard method [63]. First, using a standard cutter with 6 blades and 1 mm of separation between each one (Neurtek), a cross hatch is done, creating a 6 × 6 pattern. After that, a normalized adhesion tape (Tesa 4024) is applied over the crosscut pattern and quickly removed at an angle of 180°. The adhesion strength is categorized by visually assessing the amount of deposit removed from 5B (strongest adhesion/no film removal) to 0B (weakest adhesion/complete film removal). Adhesion experiments were reproduced in 3 samples for this adhesion assessment. Thickness characterization of the deposited layers was carried out using a Bruker Dektak XT Stylus Profiler.

## 3. Results and Discussion

### 3.1. Ink Formulation

The luminescent ink presented in this paper, named HRI-RhodB-02, takes as basis the work previously developed in our group on photoacid catalysed organic-inorganic hybrid inks for the preparation of photonic waveguides [48]. The hybrid organic-inorganic compound GPTMS is taken as main component (50 wt %), mixed with two monomers in the following proportions: 25 wt % of Epitkote 157 and 25 wt % of dPDMS. Epikote 157 is expected to polymerize with the organic part of GPTMS through its epoxy rings, while dPDMS will react with the inorganic network. Apart from this, 0.05 wt % of BYK-333 was added to control the surface tension of the ink and to improve its wetting to the substrate [49]. Additionally, 2 wt % of triarysulfonium hexafluorophophate salt is incorporated as photoacid generator to initiate the sol-gel and epoxy groups polymerization processes of the deposited ink when irradiated with UV light. Our luminescent hybrid formulation includes Rhodamine B, a luminescent chromophore that has been previously incorporated in hybrid organic-inorganic systems [54,55,56,57,58,59,60]. A 0.2 wt % of Rhodamine B was added to incorporate the luminescence functionality to the ink. 

### 3.2. Inkjet Printing

As mentioned, inkjet printing requires a highly precise control of the rheology of the ink as well as the jetting process itself. The overall process as well as the theoretical laws, based on non-dimensional numbers, that drive ink jetting and drop formation, have been thoroughly described in the literature [30,31]. These non-dimensional numbers, Reynolds (Re), Weber (We) and Ohnesorge (Oh), given by the following equations, overall describe the behaviour of the ink droplets according to their inertial forces, viscosity, kinetics and surface energy.

(1) Re=vρaη

(2) $$\mathrm{We}=\frac{v^{2}\rho a}{\gamma }$$

(3) $$\mathrm{Oh}=\frac{\sqrt{We}}{Re}=\frac{\eta }{\sqrt{\gamma \rho a}}$$

where *v* is the speed of the ink when it is leaving the nozzle, *η* is the viscosity, *γ* the surface tension, *ρ* the density and a the diameter of the printhead orifice. This theoretical framework enables to rationally understand and predict to some extent the proper jettability and deposition of the ink. Trying to gain insight in this respect for the formulated luminescent ink, its relevant properties have been characterized and these non-dimensional numbers have been calculated. Assuming that the surface tension does not significantly change in the small range from RT (26 °C) to the printhead operation temperature (32 °C), the value measured at RT, 25.9 mN/m, was taken for the calculations of the We and Oh numbers. On the other hand, density and viscosity, with a strong dependence on temperature, were measured at 32 °C, obtaining values of 1.1 g/cm^3^ and 28.5 mPas respectively.

Surface tension, viscosity and density are intrinsic values of the ink however drop velocity, that has a direct influence in We and Re numbers, depends on the printing configuration. The signal sent by the electronics of the printer, which is derived to the piezoelectric actuators of the printhead, is a critical variable that will determine the behaviour of the ink when jetting. Observation of the ink drops being ejected from the printhead nozzles by using a Dropwatcher system, enables the optimization of the voltage signal to obtain a good printability. Too low voltage can derive in the absence of printing as not enough energy is delivered to the fluid to generate a jet. On the other hand, too high voltages can cause the formation of satellites that result in inaccurate printing (Figure S1 in the Supporting information).

**Figure 1:**
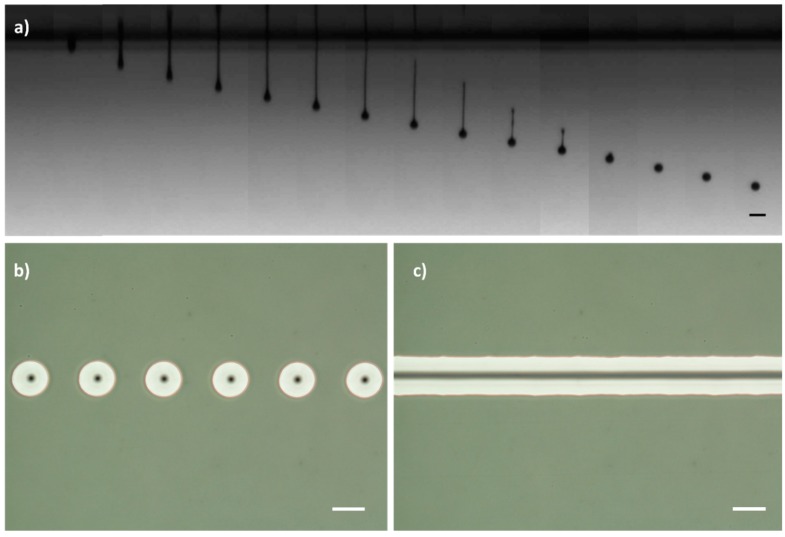
(**a**) Temporal sequence of photographs (from left to right) showing the drop formation process for the luminescent ink. The time interval between two adjacent frames is 20 µs (scale bar: 100 μm). (**b**,**c**) Phase contrast microscope images of the inkjet-printed drops of luminescent ink deposited along a line in an ozone treated glass substrate at different dpi (scale bar: 100 µm): (**b**) 120 dpi and (**c**) 360 dpi.

Figure 1a shows luminescent ink droplets ejected, in an optimized configuration, at different delay time with respect the piezoelectric actuation: An ink jet leaves the nozzle after piezo actuation. The ink filament after the leading jet front becomes narrower on time until it breaks. The tail following the main part of the flying ink drop is then retracted towards this, finally leading to a spherical drop of ink. The presence in the formulation of chain like polymeric molecules (BYK-333) favours the retraction of the tail that forms after jet rupture, toward the ink flying drop, minimizing the formation of satellite droplets [64,65]. Speed of the droplets can also be characterized by taking a picture of the same droplet at two different times using a double strobe configuration in our dropwatcher system. Average speed is estimated by taking the ratio of the flying distance and the time difference between light pulses. Figure S2 in the Supporting information shows a double strobe image of a set of luminescent ink drops with a temporal delay of 50 μs between light pulses. Through the analysis of the image, the drop velocity was estimated to be 2.5 m/s assuming no deceleration takes place in this short distance. 

Taking the values above for viscosity, surface tension and density of the ink, speed of the droplet and the diameter of nozzle as 50 μm, (as provided by the printhead manufacturer), the Reynolds, Weber and Ohnesorge numbers were calculated, giving 4.83, 13.4 and 0.75 respectively. These values are within the range that is typically defined for inkjet printable materials [30,31,66,67,68,69] and remarkably this is achieved using a solvent-free ink. The low molecular weight monomers, precursor components for the final deposit, enable the preparation of tailored formulations with appropriate properties for inkjet printing. Apart from the jettability, the luminescent ink presents good stability when stored in a closed amber flask protected from the UV light. Storage over periods longer than 4 months did not significantly change the optimum printing parameters.

In order to take advantage of the printing technology and deposit well-defined luminescent patterns, it is also necessary to control the interaction between the flying ink droplet and the receiving substrate. Due to the hybrid nature of our ink and the glass substrate initially used, specific cleaning protocols have been used to improve the wettability on glass, allowing the appropriate deposition of the ink drops and to promote the reaction of the functional groups of the ink monomers to the glass surface. Substrate treatment using UV-Ozone eliminates organic contaminants at the surface and leave the silanol groups exposed at the glass surface. These will afterwards react with the forming hydroxyl groups of the silanes, generated after UV exposure of the ink [61]. 

Over the ozone-treated surface, drops have been printed at different spacing between drops, that is, at different dots per inch (dpi). In Figure 1b, with 120 dpi, each droplet is isolated after deposition, presenting a circular shape and monodisperse size giving an indication of the reproducibility of the jetting and deposition process). If the space between drops is reduced (increasing to 360 dpi), as shown in Figure 1c, drops coalesce, leading to continuous, well-defined lines of ink on the substrate. 

Besides lines, preparation of homogeneous and continuous deposits requires a similar strategy, printing contiguous droplets that coalesce in both axis into well-defined areas. In contrast to UV-Ozone activation that easily leads to dewetted regions and faulty printing, CCVD using Pyrosil^®^ treatment was used in the search of these continuous printed regions. Through this CCVD process, a silanol porous nanolayer is created in the surface (see SEM image in Figure S3 in the Supporting information), prior to the deposition of the ink, which favours wetting. Printing on these treated surfaces with suitable dots density (e.g., 360 dpi in the substrate moving direction), led to homogenous films with uniform thickness (~4 μm).

### 3.3. Photocuring process

The effect of UV light exposure of the formulation including the luminescent dye has been studied. A thin film of luminescent HRI-RhodB-02 ink was formed between two quartz plates with a fixed gap in between (determined by the presence of spacers of fixed diameter spheres). The exact gap of the quartz cell before filling the cell with the photocurable ink was determined by interferometry, being 11 µm. Once filled by capillary action, UV-Vis absorption of the liquid thin layer was measured. The spectrum (Figure S4a in the Supporting information) shows two well-differentiated absorption peaks: First, at around 300 nm, which is assigned to the maximum absorption band of the photoacid generator (see Figure S4b of the photocurable ink without dye in the Supporting information) and a second one, at around 560 nm, corresponding to the maximum of the absorption band of the Rhodamine B dye. This dye, previously incorporated in hybrid materials, was selected due to its compatibility and solubility with the rest of components of the ink, as well as its little absorption in the UV region [70] where the photoinitiator strongly absorbs actinic radiation needed to initiate the photopolymerization reaction. Exposure of the liquid layer of our luminescent formulation to UV light irradiation (320–390 nm, 10 mW/cm^2^, 300 s) leads to a decrease of the absorption band in this UV region of the spectra, a change that is ascribed to the photoinitiator decomposition (Figure S4a in the Supporting information). A slight increase in the absorption is observed for the Rhodamine B band at 560 nm after UV exposure. Polymerization taking place in the host material can be responsible of this change in the absorption spectrum as previously reported in hybrid rhodamine B containing systems [60]. 

**Figure 2:**
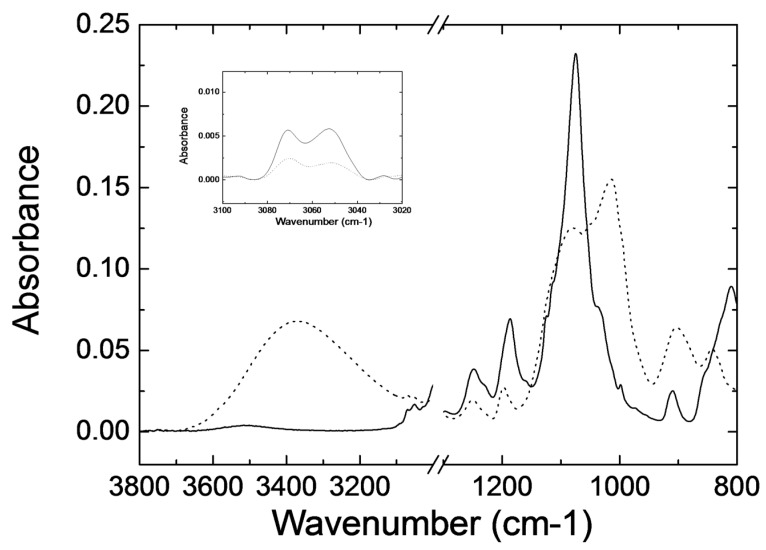
FTIR spectra of the HRI-RhodB-02 luminescent ink before (continuous line) and 10 min after UV exposure (dotted line) under atmospheric conditions. Inset shows the band of the epoxide at 3050 cm^−1^ corrected by using a linear baseline.

Macroscopically, the UV irradiation of a liquid film of photocurable formulation immediately leads to a solid layer indicating that polymerization has efficiently taken place. As mentioned, decomposition of the photoinitiator leads to acid generation that can trigger the epoxy ring-opening cationic photopolymerization as well as the photoacid-catalysed sol-gel process as described elsewhere (see Schemes S2 and S3 in the Supporting information) [48]. To study more in detail this polymerization process, FTIR spectroscopic characterization was carried out. Figure 2 shows the FTIR spectra of the HRI-RhodB-02 luminescent formulation before UV exposure and 10 min after exposure under atmospheric curing conditions. On one hand, the appearance of a broad band at 3400 cm^−1^, which is assigned to the vibration of the OH group in the Si-OH bond, indicates that the hydrolysis reaction has efficiently taken place [71]. Besides, the narrow band at 1080 cm^−1^, observed in the uncured material and corresponding to the stretching vibration assigned to the Si-O-CH_3_ group, is substituted in the cured material by a complex multipeak band, wider than the original, with maxima in the 1000–1080 cm^−1^ region, which is attributed to the stretching vibrations of the siloxane Si-O-Si bonds. All these observations confirm that the condensation reaction that leads to the inorganic network has already taken place. On the other hand, the C–H stretching band of the epoxide at 3050 cm^−1^ diminished just after (10 min. after) UV irradiation (inset Figure 2) indicating that the epoxy ring-opening reaction has taken place forming the organic polymer [72]. Despite the reaction progression, this band did not completely disappear after UV exposure. This could be ascribed to the increase of the viscosity of the system due to polymerization. As the reaction progresses the diffusion of the photoacid generator and the monomers is more and more limited thus favouring the presence of unreacted epoxy groups [73]. This was further supported by the fact that an homologous model luminescent formulation comprising GPTMS as only reactive monomer, thus eliminating the DPDMS and EPIKOTE157 crosslinkers, showed complete depletion of the C-H stretching band of the epoxide at 3050 cm^−1^, 10 min. after UV irradiation (see Figure S5 and detailed composition of this model formulation in the Supporting information). The lower crosslinking density in this model system allows better monomer diffusion to complete the epoxy polymerization reaction.

FTIR spectra of films of the cured photopolymerizable ink were recorded 10 min and 24 h after the irradiation to assess the evolution of the system in darkness (Figure S6 in the Supporting information). Small differences in the 1000–1080 cm^−1^ region were appreciated between the spectra at 10 min and 24 h, indicating that hydrolysis and condensation have significantly taken place right after the first 10 min for samples cured under these ambient atmospheric conditions. The spectra do not qualitatively evolve after 24 h, as followed over one month. Overall it can be concluded that the presence of this percentage of this luminescent dye (0.2 wt %) in the ink does not preclude the photoreaction to proceed. As in previously studied systems, without the luminescent dye, the two polymerization processes, cationic ring-opening polymerization of the epoxide groups and the hydrolysis and condensation of the silanes, take place simultaneously after the light excitation of the photoacid generator.

**Figure 3:**
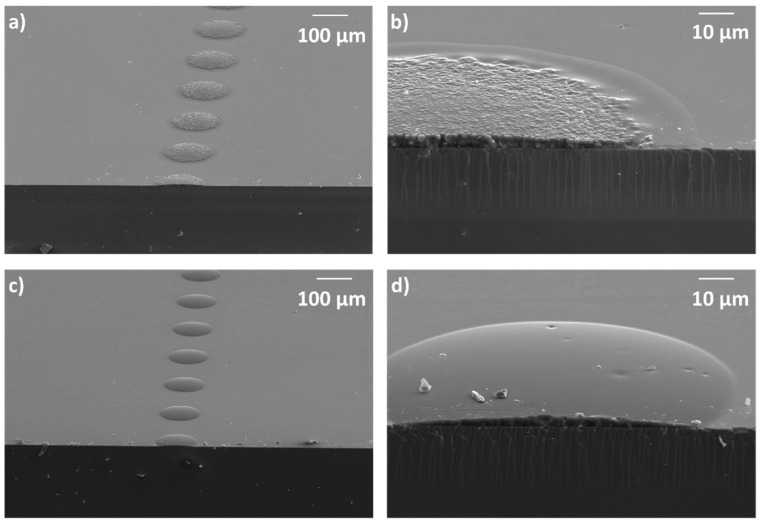
SEM morphology observed at different magnifications of (**a**) isolated inkjet-printed droplets and (**b**) fractured cross section of one droplet, cured in ambient atmospheric conditions. SEM morphology observed at different magnifications of (**c**) isolated inkjet-printed droplets and (**d**) fractured cross section of one droplet, cured under mild vacuum conditions (100 mBar).

Figure 3a,b shows SEM microscopy images of droplets deposited under optimum jetting conditions and cured in ambient atmosphere (at 26 °C and relative humidity between 30%–40%). The SEM morphology of deposited ink droplets presents inhomogeneous structures in the surface after curing, indicating phase segregation [48]. This effect appears during the curing process in these hybrids multicomponent inks and can be due to the different kinetics of the organic and inorganic parts during their polymerization. The presence of atmospheric water, needed for the hydrolysis step, causes acceleration in the formation of the inorganic network, while the influence of water presence in the cationic ring-opening polymerization reaction of the organic network is less pronounced [74]. This can be used to adjust the relative polymerization rates of the two networks. To avoid this phenomenon, the luminescent ink has been exposed to UV light irradiation at mild vacuum conditions (100 mBar), reducing the amount of available atmospheric water. Figure 3c,d shows SEM microscopy images of isolated deposited droplets cured under these mild vacuum conditions. The cured droplets present a homogeneous, scattering-free, surface morphology. In this last case, due to the reduction of the humidity in the atmosphere, both organic and inorganic network could progress uniformly without phase segregation, leading to a smooth surface of the cured droplet. FTIR experiments were also carried out on a sample cured under mild vacuum conditions to reduce the water content. The spectrum taken 24 h after UV irradiation (Figure S7 in the Supporting information) in this sample did not qualitatively differ from the one taken in a homologous sample cured in ambient atmospheric conditions despite the important differences in morphology. In all cases, regardless of the curing atmosphere, no evolution of the FTIR spectrum was observed for periods of time longer than 24 h and therefore all the films in this work were allowed to evolve in darkness for at least 24 h before any subsequent characterization to ensure completion of the polymerization process.

For subsequent studies, we focused in the curing conditions under mild vacuum, leading to non-segregated morphology and therefore homogenous and scattering-free printed elements more suitable for optical applications. The hardness and Young modulus, determined by nanoindentation in 4 µm thick films, were 0.26 and 4.3 GPa, respectively. These values are similar to those measured for homologous deposits without the luminescent dye [48]. Excellent adhesion to the substrate was also found for the cured luminescent ink as can already be anticipated from the SEM images of the fractured cured isolated drops (Figure 3). To assess the adhesion of the cured ink to the glass substrate, a cross-cut and tape test according to the standard ASTM 3359 was carried out. The samples were evaluated in detail with optical microscope after cross cutting the films and removing the tape. As can be seen in Figure S8 in the Supporting information, the cross cut areas do not show significant damages and only slightly detached flakes can be appreciated where the blade has passed, however delamination is below 5% of the affected area resulting in a classification of 4B-5B binding strength according to the defined standard. 

### 3.4. Luminescent Properties of the Printed Films

**Figure 4:**
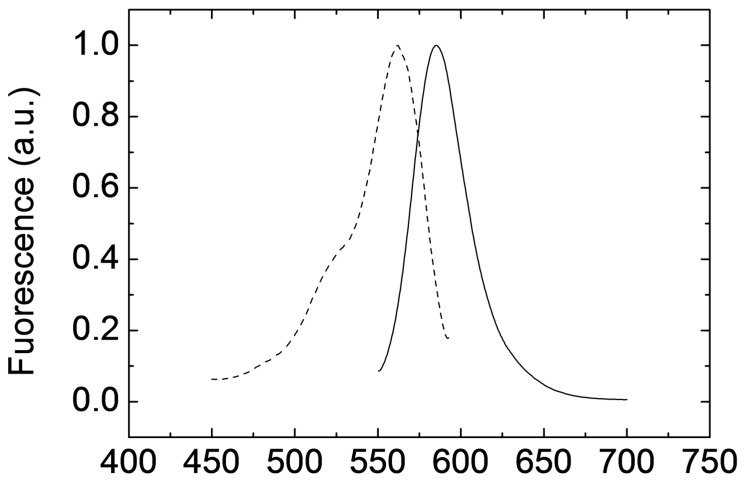
Photoluminescence emission (continuous line) and excitation (dashed line) spectra of deposited films of HRI-RhodB-02 cured at mild vacuum. Photoluminescence emission spectrum is taken with excitation at 530 nm and excitation spectrum is measured at 610 nm.

Deposits of luminescent ink with homogeneous thickness were prepared using the previously described process (inkjet-printed film cured under mild vacuum conditions) and their optical properties were analysed afterwards. Photoluminescence emission and excitation spectra were measured and are shown in Figure 4. It can be seen that the maximum of the excitation spectrum is reached at 562 nm that coincides with the maximum of the absorption spectrum (Figure S4 in the Supporting information). The maximum of emission is red-shifted to 585 nm. These optical properties are given by the excitation of Rhodamine-B dye as the rest of the matrix components present no light absorption in this wavelength range [48].

### 3.5. Digital Patterning on Different Substrates 

**Figure 5:**
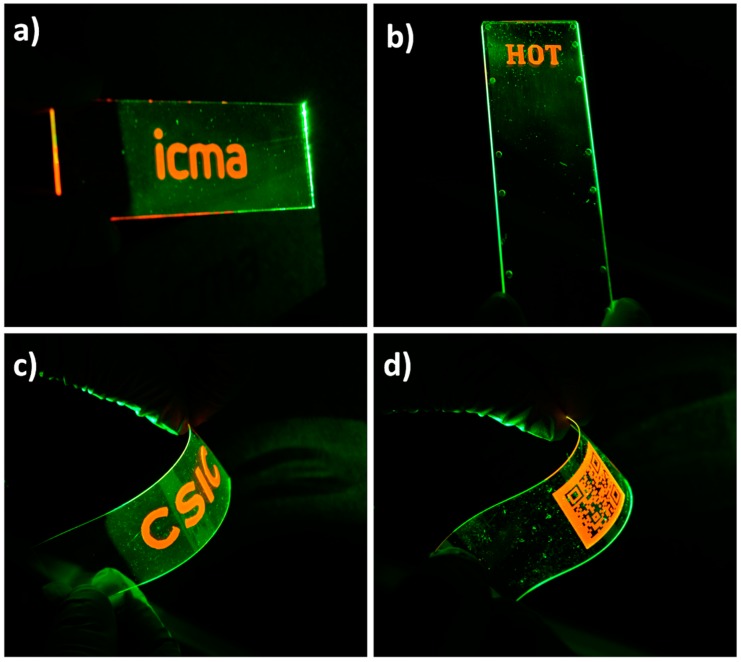
Luminescent prints on different substrates (7.5 cm × 2.5 cm) shown under green light excitation. Luminescent marks on (**a**) glass, (**b**) rigid COP and (**c**) flexible ITO coated PET. (**d**) Luminescent QR code printed on flexible COP.

Fine control of the spatial positioning provided by inkjet printing enables the preparation of well-defined luminescent patterns, such as the marks or the QR codes shown in Figure 5. Besides the printing of luminescent patterns on glass (Figure 5a), the ink has also been applied on top of thermoplastic substrates such as COP, a material typically used in the preparation of microfluidic devices [75] to put in value the versatility of these photoacid catalysed systems. As an example, Figure 5b shows a print of luminescent ink on top of a rigid COP microscope slide. Printing has also been performed on top of flexible substrates. Figure 5c shows an image printed with the luminescent ink on top of a flexible PET foil coated with ITO. Figure 5d presents a luminescent QR code printed on a flexible COP foil. The orange emitted light from this QR code, when excited with green light, can be immediately recognized by standard QR code recognition software of a conventional mobile phone (Camera app from iPhone 8 by Apple, iOS 12.1.4). Reading of the code is done through a long-pass red filter (Cut-On Wavelength: 600 nm) to ensure that the emitted light is use for code recognition. Neither detachment, nor cracking of the luminescent marks was observed against sample bending, as shown in Figure 5c,d, demonstrating good mechanical stability of the deposited marks also on these flexible substrates. All these demonstrators prove the versatility of these luminescent organic-inorganic single-step curing inks to generate well-defined luminescent functional marks on a variety of industrially relevant substrates.

## 4. Conclusions

A luminescent ink based on photoacid catalysed organic-inorganic hybrid formulations has been prepared. The incorporation of a small percentage of Rhodamine-B in photopolymerizable formulations containing monomers with epoxy and silane functionalities as well as photoacid generators, led to a jettable, photocurable ink. UV light exposure triggered the cationic ring-opening polymerization reaction of the epoxy groups and, in the presence of atmospheric water, the hydrolysis and condensation of the inorganic network, overall leading to a crosslinked organic-inorganic polymeric material. Control of the curing conditions led to deposits presenting excellent adhesion to glass, reduced light scattering and luminescence, all this through a simple one-step curing process without the need of any post baking steps that could degrade the luminescent dye. Overall, the present photoacid catalysed strategy allows, through an adequate selection of monomers and functional additives, the preparation of functional inks with tailored optical properties. The photolatency of the presented system, that delays the hydrolysis and condensation reactions in the absence of actinic light, favours the long-term stability of the ink properties overcoming problems arising in inks based on conventional sol-gel processes. Digital patterning of these inks leads to luminescent patterns that can be deposited on different industrially relevant substrates such as glass, PET or COP demonstrating the suitability of these photoacid catalysed organic-inorganic hybrid inks for the preparation of optical elements and devices.

## Supplementary Materials

The following are available online at https://www.mdpi.com/2073-4360/11/3/430/s1.

## References

[1] Kobayashi S., Mikoshiba S., Lim S. (2009). Flat Panel Display Manufacturing. LCD Backlights.

[2] Konstantatos G., Sargent E.H. (2013). Colloidal Quantum Dot Optoelectronics and Photovoltaics.

[3] Debije M.G., Verbunt P.P.C. (2012). Thirty Years of Luminescent Solar Concentrator Research: Solar Energy for the Built Environment. Adv. Energy Mater..

[4] Liang R.-Q., Li W., Li Y., Tan C.-Y., Li J.-X., Jin Y.-X., Ruan K.-C. (2005). An oligonucleotide microarray for microRNA expression analysis based on labeling RNA with quantum dot and nanogold probe. Nucleic Acids Res..

[5] Brites C.D.S., Lima P.P., Silva N.J.O., Millan A., Amaral V.S., Palacio F., Carlos L.D. (2010). A luminescent molecular thermometer for long-term absolute temperature measurements at the nanoscale. Adv. Mater..

[6] Baride A., Meruga J.M., Douma C., Langerman D., Crawford G., Kellar J.J., Cross W.M., May P.S. (2015). A NIR-to-NIR upconversion luminescence system for security printing applications. RSC Adv..

[7] You M., Lin M., Wang S., Wang X., Zhang G., Hong Y., Dong Y., Jin G., Xu F. (2016). Three-dimensional quick response code based on inkjet printing of upconversion fluorescent nanoparticles for drug anti-counterfeiting. Nanoscale.

[8] Binnemans K. (2009). Lanthanide-based luminescent hybrid materials. Chem. Rev..

[9] Bao B., Li M., Li Y., Jiang J., Gu Z., Zhang X., Jiang L., Song Y. (2015). Patterning fluorescent quantum dot nanocomposites by reactive inkjet printing. Small.

[10] Shirasaki1 Y., Supran G.J., Bawendi M.G., Bulovic V. (2013). Emergence of colloidal quantum-dot light-emitting technologies. Nat. Photon..

[11] Guillou O., Daiguebonne C., Calvez G., Bernot K. (2016). A long journey in lanthanide chemistry: From fundamental crystallogenesis studies to commercial anticounterfeiting taggants. Acc. Chem. Res..

[12] Frath D., Massue J., Ulrich G., Ziessel R. (2014). Luminescent materials: Locking p-conjugated and heterocyclic ligands with boron(III). Angew. Chem. Int. Ed..

[13] Wang F., Xie Z., Zhang B., Liu Y., Yang W., Liu C.Y. (2014). Down- and up-conversion luminescent carbon dot fluid: Inkjet printing and gel glass fabrication. Nanoscale.

[14] Deng C., Yang Z., Zheng Z., Liu N., Ling J. (2015). Photoluminescent nanoparticles in water with tunable emission for coating and ink-jet printing. J. Mater. Chem. C.

[15] Lee W.-H., Park Y.D. (2017). Inkjet Etching of Polymers and Its Applications in Organic Electronic Devices. Polymers.

[16] Homola T., Shekargoftar M., Dzik P., Krumpolec R., Durasova Z., Vesely M., Cernak M. (2017). Low-temperature (70 °C) ambient air plasma-fabrication of inkjet-printed mesoporous TiO2 flexible photoanodes. Flex. Print. Electron..

[17] Ma S., Ribeiro F., Powell K., Lutian J., Møller C., Large T., Holbery J. (2015). Fabrication of Novel Transparent Touch Sensing Device via Drop-on-Demand Inkjet Printing Technique. ACS Appl. Mater. Interfaces.

[18] Sun J.Z., Guo Y.Z., Cui B., Chu F.Q., Li H.Z., Li Y., He M., Ding D., Liu R.P., Li L.H. (2018). Inkjet printing bendable circuits based on an oil-water interface reaction. Appl. Surf. Sci..

[19] Sun J.Z., Yun C., Cui B., Li P., Liu G., Wang X., Chu F. (2018). A Facile Approach for Fabricating Microstructured Surface Based on Etched Template by Inkjet Printing Technology. Polymers.

[20] Sun J.Z., Cui B., Chu F.Q., Yun C.H., He M., Li L.H., Song Y.L. (2018). Printable nanomaterials for the fabrication of high-performance supercapacitors. Nanomaterials.

[21] Bollgruen P., Gleissner U., Wolfer T., Megnin C., Mager D., Overmeyer L., Korvink J.G., Hanemann T. (2016). Ink-jet printed fluorescent materials as light sources for planar optical waveguides on polymer foils. Opt. Eng..

[22] Zhang H.-B., Liu M., Lei X., Wen T., Zhang J. (2014). Digital controlled luminescent emission via patterned deposition of lanthanide coordination compounds. ACS Appl. Mater. Interfaces.

[23] daLuz L.L., Milani R., Felix J.F., Ribeiro I.R.B., Talhavini M., Neto B.A.D., Chojnacki J., Rodrigues M.O., Júnior S.A. (2015). Inkjet printing of lanthanide−organic frameworks for anti-counterfeiting applications. ACS Appl. Mater. Interfaces.

[24] Robin M., Kuai W., Amela-Cortes M., Cordier S., Molard Y., Mohammed-Brahim T., Jacques E., Harnois M. (2015). Epoxy based ink as versatile material for inkjet-printed devices. ACS Appl. Mater. Interfaces.

[25] Haverinen H.M., Myllyla R.A., Jabbour G.E. (2009). Inkjet printing of light emitting quantum dots. Appl. Phys. Lett..

[26] Andres J., Hersch R.D., Moser J.-E., Chauvin A.-S. (2014). A new anti-counterfeiting feature relying on invisible luminescent full color images printed with lanthanide-based inks. Adv. Funct. Mater..

[27] You M., Zhong J., Hong Y., Duan Z., Lin M., Xu F. (2015). Inkjet printing of upconversion nanoparticles for anti-counterfeit applications. Nanoscale.

[28] Wang Y.-M., Tian X.-T., Zhang H., Yang Z.-R., Yin X.-B. (2018). Anticounterfeiting quick response code with emission color of invisible metal—Organic frameworks as encoding information. ACS Appl. Mater. Interfaces.

[29] Singh M., Haverinen H.M., Dhagat P., Jabbour G.E. (2010). Inkjet printing-process and its applications. Adv. Mater..

[30] Derby B. (2010). Inkjet printing of functional and structural materials: Fluid property requirements, feature, stability, and resolution. Annu. Rev. Mater. Res..

[31] Alaman J., Alicante R., Pena J., Sanchez-Somolinos C. (2016). Inkjet printing of functional materials for optical and photonic applications. Materials.

[32] Coenen M.J.J., Slaats T.M.W.L., Eggenhuisen T.M., Groen P. (2015). Inkjet printing the three organic functional layers of two-colored organic light emitting diodes. Thin Solid Films.

[33] Samusjew A., Kratzer M., Moser A., Teichert C., Krawczyk K.K., Griesser T. (2017). Inkjet Printing of Soft, Stretchable Optical Waveguides through the Photopolymerization of High-Profile Linear Patterns. ACS Appl. Mater. Interfaces.

[34] Kim J.Y., Martin-Olmos C., Baek N.S., Brugger J. (2013). Simple and easily controllable parabolic-shaped microlenses printed on polymeric mesas. J. Mater. Chem. C.

[35] Descombes L.J., Cadarso V.J., Schleunitz A., Grutzner S., Klein J.J., Brugger J., Schift H., Grutzner G. (2015). Organic-inorganic-hybrid-polymer microlens arrays with tailored optical characteristics and multi-focal properties. Opt. Express.

[36] Eggenhuisen T.M., Galagan Y., Biezemans A.F.K.V., Slaats T.M.W.L., Voorthuijzen W.P., Kommeren S., Shanmugam S., Teunissen J.P., Hadipour A., Verhees W.J.H. (2015). High efficiency, fully inkjet printed organic solar cells with freedom of design. J. Mater. Chem. A.

[37] Tekin E., Smith P.J., Hoeppener S., van den Berg A.M.J., Susha A.S., Rogach A.L., Feldmann J., Schubert U.S. (2007). Inkjet printing of luminescent CdTe nanocrystal–polymer composites. Adv. Funct. Mater..

[38] Tekin E., de Gans B.-J., Schubert U.S. (2004). Ink-jet printing of polymers from single dots to thin film libraries. J. Mater. Chem..

[39] Jacot-Descombes L., Gullo M.R., Cadarso V.J., Brugger J. (2012). Fabrication of epoxy spherical microstructures by controlled drop-on-demand inkjet printing. J. Micromech. Microeng..

[40] Bollgruen P., Gleissner U., Megnin C., Mager D., Korvink J., Hanemann T. (2016). Ink-jet printing of host-guest systems based on acrylates with fluorescent dopants. SPIE Proc..

[41] Serra A., Ramis X., Fernández-Francos X. (2016). Epoxy sol-gel hybrid thermosets. Coatings.

[42] Sanchez C., Lebeau B., Chaput F., Boilot J.-P. (2003). Optical properties of functional hybrid organic-inorganic nanocomposites. Adv. Mater..

[43] Houbertz R., Frohlich L., Popall M., Streppel U., Dannberg P., Bräuer A., Serbin J., Chichkov B.N. (2003). Inorganic-Organic Hybrid Polymers for Information Technology: From Planar Technology to 3D Nanostructures. Adv. Eng. Mater..

[44] Sanchez C., Belleville P., Popall M., Nicole L. (2011). Applications of advanced hybrid organic–inorganic nanomaterials: From laboratory to market. Chem. Soc. Rev..

[45] Parola S., Julian-Lopez B., Carlos L.D., Sanchez C. (2016). Optical properties of hybrid organic-inorganic materials and their applications. Adv. Funct. Mater..

[46] Sriramulu D., Turaga S.P., Yi A.X., Bettiol A.A., Valiyaveettil S. (2016). Synthesis, characterization and application of luminescent silica nanomaterials. J. Mater. Chem. C.

[47] Hong Y., Chen Z., Trofimov A.A., Lei J., Chen J., Yuan L., Zhu W., Xiao H., Xu D., Jacobsohn L.G. (2017). Direct inkjet printing of miniaturized luminescent YAG:Er3+ from sol-gel precursor. Opt. Mater..

[48] Alamán J., López-Valdeolivas M., Alicante R., Medel F.J., Silva-Treviño J., Peña J.I., Sánchez-Somolinos C. (2018). Photoacid catalyzed organic–inorganic hybrid inks for the manufacturing of inkjet-printed photonic devices. J. Mater. Chem. C.

[49] Chemtob A., Versace D.-L., Belon C., Croutxe-Barghorn C., Rigolet S. (2008). Concomitant organic-inorganic UV-curing catalyzed by photoacids. Macromolecules.

[50] Chemtob A., Peter M., Belon C., Dietlin C., Croutxé- Barghorn C., Vidal L., Rigolet S. (2010). Macroporous organosilica films via a template-free photoinduced sol–gel process. J. Mater. Chem..

[51] Croutxé-Barghon C., Belon C., Chemtob A. (2010). Polymerization of hybrid sol-gel materials catalyzed by photoacids generation. J. Photopolym. Sci. Technol..

[52] Danzebrink R., Aegerter M.A. (1999). Deposition of micropatterned coating using an ink-jet technique. Thin Solid Films.

[53] Danzebrink R., Aegerter M.A. (2001). Deposition of optical microlens arrays by ink-jet processes. Thin Solid Films.

[54] Fujii T., Isbii A., Anpo M. (1990). Absorption and fluorescence spectra of rhodamine B molecules encapsulated in silica gel networks and their thermal stability. J. Photochem. Photobiol. A.

[55] Severin-Vantilt M.M.E., Oomen E.W.J.L. (1993). The incorporation of Rhodamine B in silica sol-gel layers. J. Non-Cryst. Solids.

[56] Zareba-Grodz I., Pazik R., Hermanowicz K., Strek W., Maruszewski K. (2006). Preparation and optical properties of hybrid coatings based on epoxy-modified silane and rhodamine B. J. Lumin..

[57] del Monte F., Levy D. (1998). Formation of fluorescent Rhodamine B J-dimers in sol-gel glasses induced by the adsorption geometry on the silica surface. J. Phys. Chem. B.

[58] Schulz-Ekloff G., Wöhrle D., van Duffel B., Schoonheydt R.A. (2002). Chromophores in porous silicas and minerals: Preparation and optical properties. Microporous Mesoporous Mater..

[59] Nedelcev T., Racko D., Krupa I. (2008). Preparation and characterization of a new derivative of rhodamine B with an alkoxysilane moiety. Dyes Pigm..

[60] Negishi N., Fujino M., Yamashita H., Fox M.A., Anpo M. (1994). Photophysical properties and photochemical stability of Rhodamine B encapsulated in S1O2 and Si-Ti binary oxide matrices by the sol-gel method. Langmuir.

[61] Fink C.K., Nakamura K., Ichimura S., Jenkins S.J. (2009). Silicon oxidation by ozone. J. Phys. Condens. Matter.

[62] Schuhmacher B., Muschenborn W., Stratmann M., Schultrich B., Klages C.-P., Kretschmer M., Seyfert U., Forster F., Tiller H.-J. (2001). Novel coating systems and surface technologies for continuous processing of steel sheet. Adv. Eng. Mater..

[63] Standard ASTM D3359-02 (2002). Standard Test Methods for Measuring Adhesion by Tape Test.

[64] Hoath S.D., Hutchings I.M., Martin G.D., Tuladhar T.R., Mackley M.R., Vadillo D. (2009). Links between ink rheology, drop-on-demand jet formation, and printability. J. Imaging Sci. Technol..

[65] Hoath S.D., Vadillo D.C., Harlen O.G., McIlroy C., Morrison N.F., Hsiao W.-K., Tuladhar T.R., Jung S., Martin G.D., Hutchings I.M. (2014). Inkjet printing of weakly elastic polymer solutions. J. Non-Newton. Fluid Mech..

[66] Derby B., Reis N., Seerden K.A.M., Grant P.S., Evans J.R.G. (2000). Freeform fabrication of ceramics by hot-melt ink-jet printing. MRS Proc..

[67] Duineveld P.C., de Kok M.M., Buechel M., Sempel A., Mutsaers K.A., van de Weijer P., Camps I.G.J., van de Biggelaar T., Rubingh J.-E.J.M., Haskal E.I. (2002). Ink-jet printing of polymer light-emitting devices. Proc. SPIE.

[68] Stow C.D., Hadfield M.G. (1981). An experimental investigation of fluid flow resulting from the impact of a water drop with an unyielding dry surface. Proc. R. Soc. Lond. A Math. Phys. Eng. Sci..

[69] Bhola R., Chandra S. (1999). Parameters controlling solidification of molten wax droplets falling on a solid surface. J. Mater. Sci..

[70] Bartasun P., Cieslinski H., Bujacz A., Wierzbicka-Wos A., Kur J. (2013). A study on the interaction of Rhodamine B with Methylthioadenosine Phosphorylase protein sourced from an antarctic soil metagenomic library. PLoS ONE.

[71] De Paz H., Chemtob A., Croutxé-Barghorn C., Le Nouen D., Rigolet S. (2012). Insights into Photoinduced Sol-Gel Polymerization: An in Situ Infrared Spectroscopy Study. J. Phys. Chem. B.

[72] Chemtob A., Ni L., Dietlin C., Croutxé-Barghorn C., Kitzmann P., Brogly M., Vidal L. (2012). Spontaneous photoinduced formation of hybrid polymer films with functionalized macroporous patterns. Surf. Coat. Technol..

[73] Keller S., Blagoi G., Lillemose M., Haefliger D., Boisen A. (2008). Processing of thin SU-8 films. J. Micromech. Microeng..

[74] Sangermano M., Razza N., Crivello J.V. (2014). Cationic UV-Curing: Technology and Applications. Macromol. Mater. Eng..

[75] Nunes P.S., Ohlsson P.D., Ordeig O., Kutter J.P. (2010). Cyclic olefin polymers: Emerging materials for lab-on-a-chip applications. Microfluid. Nanofluid..

