# Supplementary material for: Digital Luminescence Patterning via Inkjet Printing of a Photoacid Catalysed Organic-Inorganic Hybrid Formulation
Source: Polymers (Basel). 2019 Mar 6;11(3):430. doi: 10.3390/polym11030430 (PMC6473484; doi:10.3390/polym11030430)
Supplement: Supplementary file 1 [file polymers-11-00430-s001.pdf]

polymers

MDPI

Supplementary information

Digital luminescence patterning via inkjet printing of a photoacid catalyzed organic-inorganic hybrid formulation

Jorge Alamán[a, b], María López-Valdeolivas[a], Raquel Alicante[a], Jose Ignacio Peña[c], and Carlos Sánchez-Somolinos[a, d]*

a Instituto de Ciencia de Materiales de Aragón (ICMA), CSIC-Universidad de Zaragoza, Departamento de Física de la Materia Condensada, Zaragoza, Spain

b BSH Electrodomésticos España, S.A., Polígono Industrial de PLA-ZA, Ronda del Canal Imperial de Aragón, 18-20, 50197 Zaragoza, Spain

c Instituto de Ciencia de Materiales de Aragón (ICMA), CSIC-Universidad de Zaragoza, Departamento de Ciencia y Tecnología de Materiales y Fluidos, Zaragoza, Spain

d CIBER in Bioengineering, Biomaterials and Nanomedicine (CIBER-BBN), Spain

* Correspondence author: carlos.s@csic.es

Telephone: +34 876 55 3770

Supplementary Materials

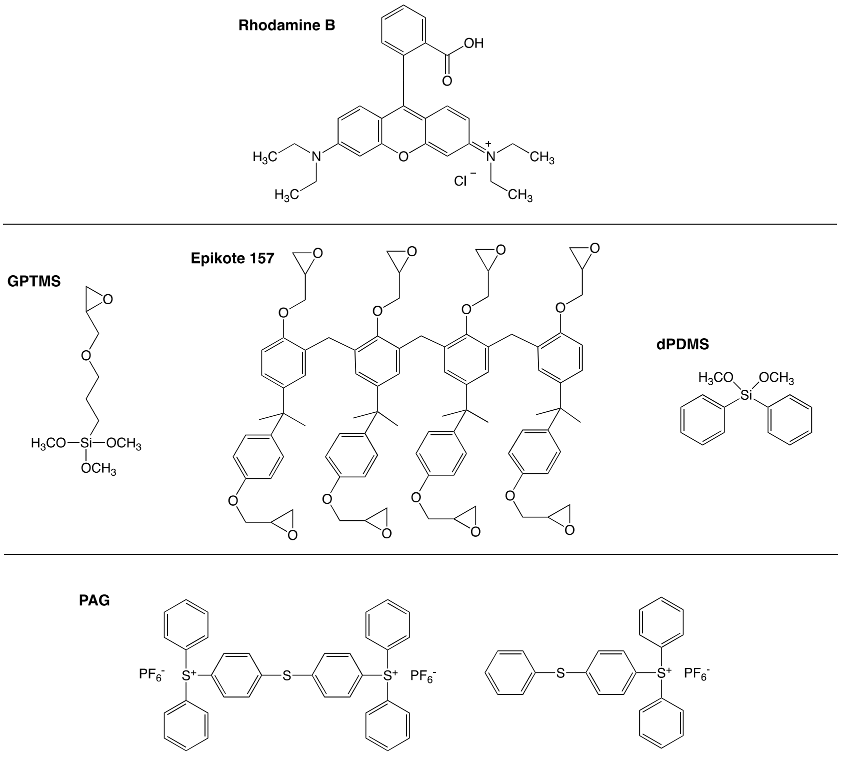

Scheme S1. Chemical structure of the main components of the inks. Rhodamine B, GPTMS (3-glycidoxypropyltrimethoxysilane), Epikote 157, dPDMS (Dimethoxydiphenylsilane) and PAG (triarylsulfonium hexafluorophosphate salts, 50% in propylene carbonate).

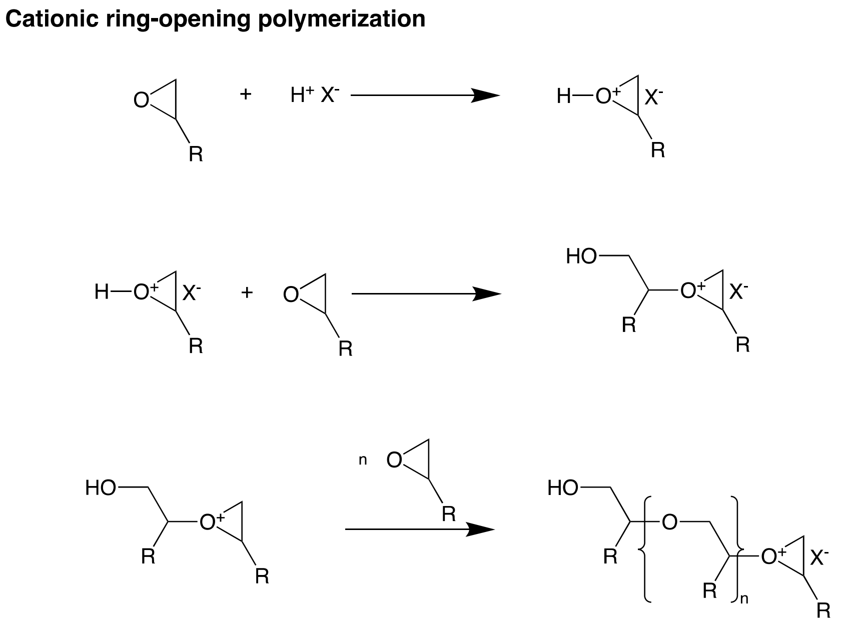

Scheme S2. Cationic ring-opening polymerization of epoxy monomers.

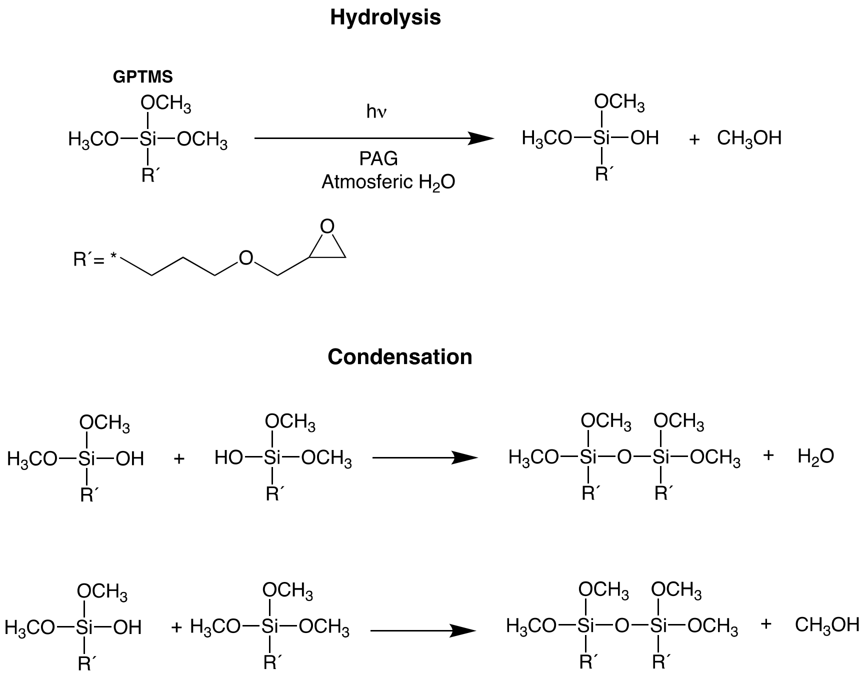

Scheme S3. Hydrolysis and condensation steps of the photoinduced sol-gel process for GPTMS monomer.

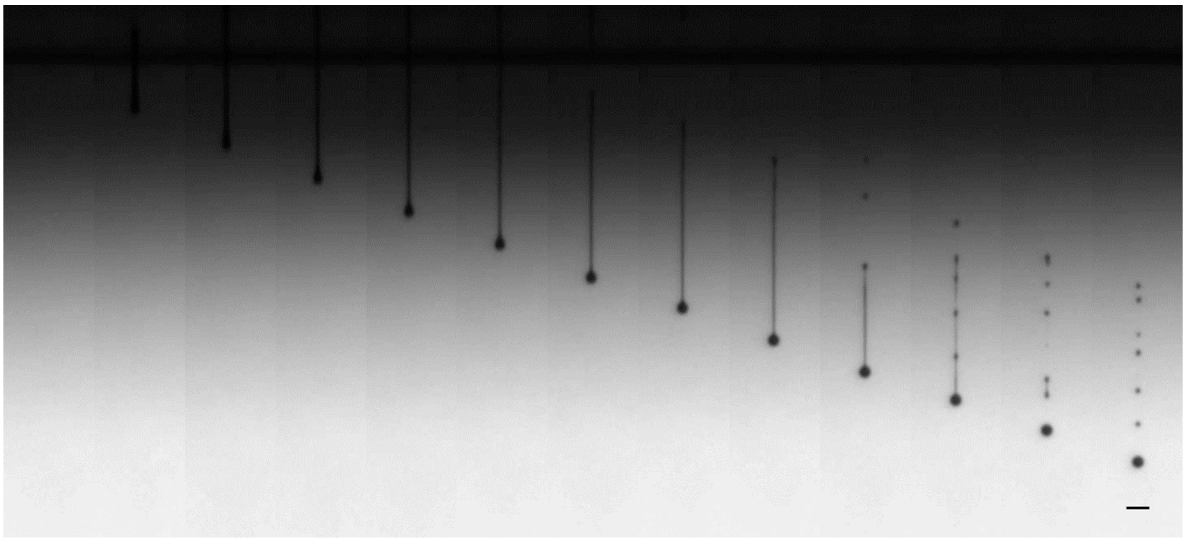

Figure S1. Temporal sequence of photographs (from left to right) showing the drop formation process for the luminescent ink with excess of energy in the jet. The time interval between two adjacent frames is 24 μs (scale bar: 100 μm).

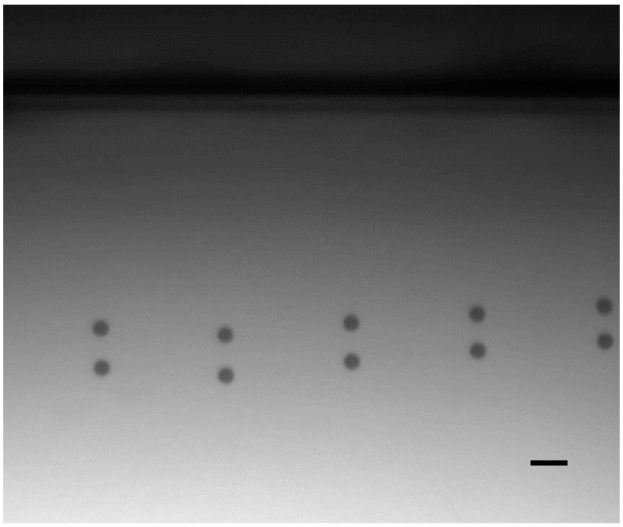

Figure S2. Double strobe image of a set of ejected droplets. The five droplets at the upper row are the same as the ones in the lower row. The five droplets are imaged twice with a delay of 50 μs. The distance between the images corresponding to the same droplets are used for the calculation of speed (scale bar: 100 μm).

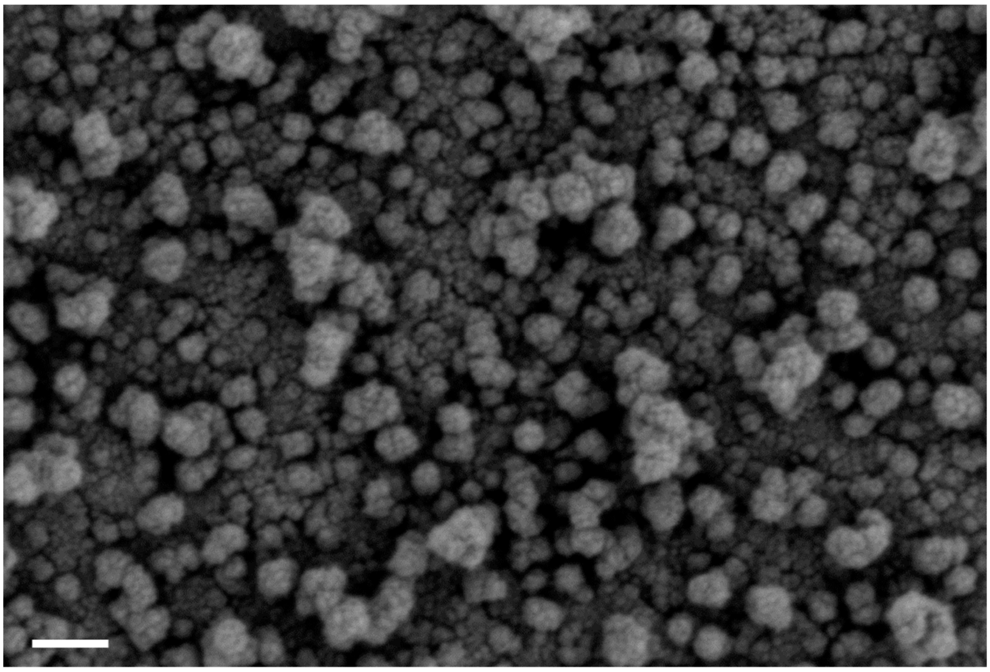

Figure S3. SEM image of a Pyrosil treated glass surface (scale 100 nm).

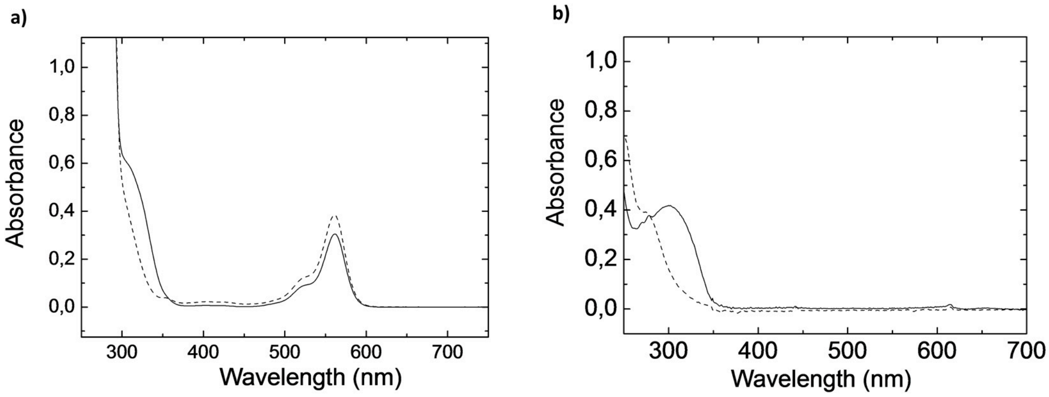

Figure S4. UV-Vis absorption spectra of (a) a thin film of luminescent ink and (b) the same formulation without dye, between quartz glass plates (11 μm gap) before (continuous line) and after UV irradiation (dashed line).

Model-RhodB-02 ink

A model luminescent system, named Model-RhodB-02, based of the organic-inorganic hybrid GPTMS as only reactive monomer, has been prepared as a reference system to follow the photocuring reaction. A small percentage of 2 wt %. of triarylsulfonium hexafluorophosphate salts is incorporated to the formulation as a PAG. Additionally 0.05 wt % of BYK-333 and 0,2 wt % of Rhodamine B were added.

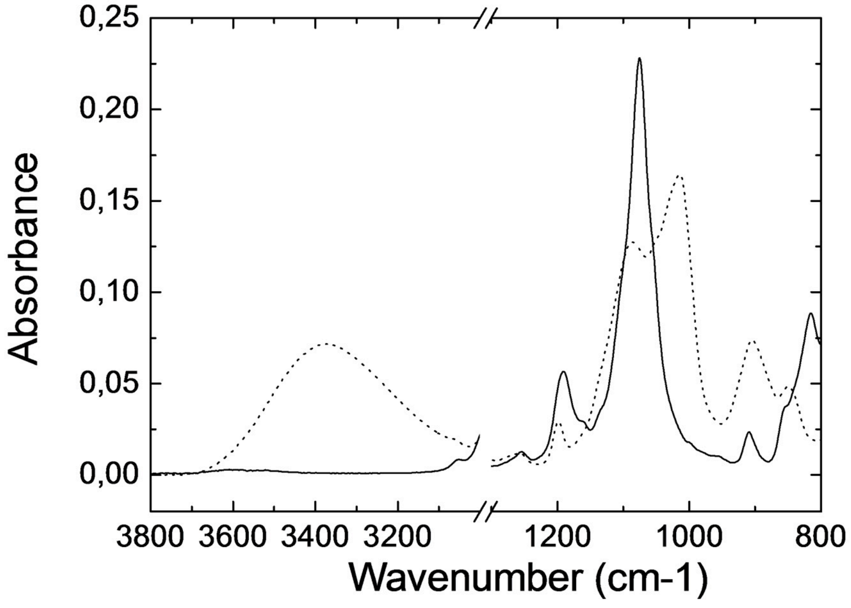

Figure S5. FTIR spectra of Model-RhodB-02 ink before (continuous line) and 10 minutes after UV exposure (dotted line) under atmospheric conditions.

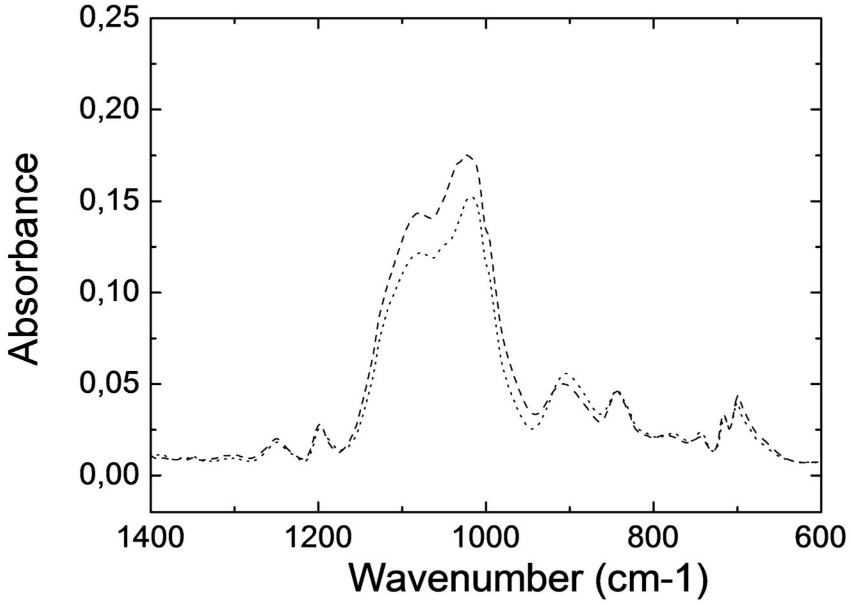

Figure S6. FTIR spectra of a thin film of HRI-RhodB-02 luminescent formulation 10 minutes (dots line) and 24 hours after curing (dashed line) cured under atmospheric conditions.

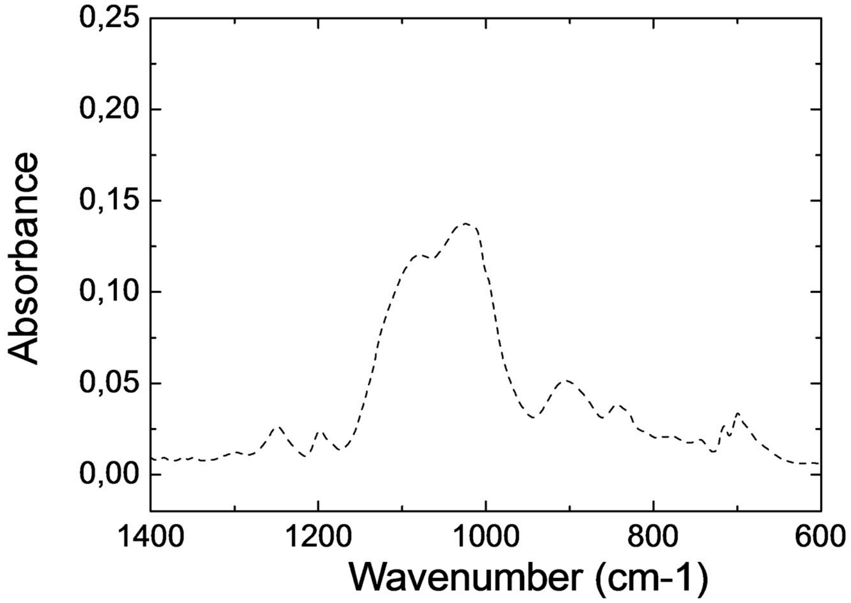

Figure S7. FTIR spectrum of a thin film of HRI-RhodB-02 luminescent formulation 24 hours after curing (dashed line) under mild vacuum conditions (100 mBar).

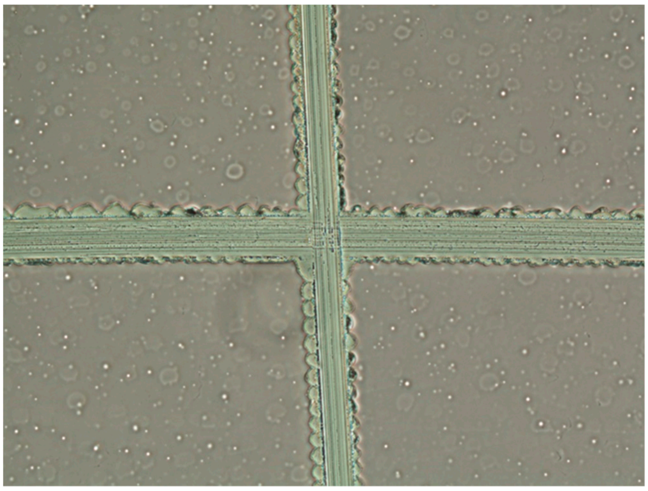

Figure S8. Optical microscope images of deposited films cured under mild vacuum conditions after the ASTM 3359 adhesion test. Film thickness: 4 μm.
